# SPECT in the Kleine–Levin Syndrome, a Possible Diagnostic and Prognostic Aid?

**DOI:** 10.3389/fneur.2014.00178

**Published:** 2014-09-23

**Authors:** Patrick Vigren, Maria Engström, Anne-Marie Landtblom

**Affiliations:** ^1^Division of Neurology, Department of Clinical and Experimental Medicine, Faculty of Health Sciences, Linköping University, Linköping, Sweden; ^2^Department of Neurology, County Council of Östergötland, Linköping, Sweden; ^3^Division of Radiological Sciences, Department of Medical and Health Sciences (IMH), Faculty of Health Sciences, Linköping University, Linköping, Sweden; ^4^Center of Medical Image Science and Visualization (CMIV), Linköping University, Linköping, Sweden

**Keywords:** single photon emission tomography, sleep disorders, brain perfusion, Kleine–Levin syndrome, sleep

## Abstract

**Introduction:** Kleine–Levin syndrome (KLS) is a rare syndrome of periodic hypersomnia and behavioral and cognitive symptoms based on clinical criteria. In the setting of differential diagnosis of hypersomnia disorders, an objective diagnostic aid is desirable. A promising modality is single photon emission computed tomography (SPECT). As intraepisodal investigations are difficult to perform, an interepisodal investigation would be very helpful. Another aim of the study was to correlate SPECT findings to prognosis.

**Methods and Materials:** Twenty-four KLS-patients were categorized as severe or non-severe based on clinical characteristics. The clinical characteristics were analyzed in relation to SPECT-examinations performed between hypersomnia periods (interepisodal) or after remission, as a clinical routine investigation.

**Results:** Forty-eight percent of the KLS-patients have hypoperfusion in the temporal or fronto-temporal regions. In patients that have undergone remission, 56% show that pattern. There were no specific findings related to prognosis.

**Discussion/Conclusion:** SPECT might be a diagnostic aid, in a setting of hypersomnia experience. With a sensitivity of 48%, interepisodal SPECT alone cannot be used for diagnosing KLS.

## Introduction

The Kleine–Levin syndrome (KLS) is a rare disorder of periodic hypersomnia and associated symptoms. The diagnosis is based on clinical criteria according to the American Academy of Sleep Medicine Classification (2005), including the cardinal symptom of periodic hypersomnia and behavioral and cognitive disturbances (1). The rarity of the syndrome, in combination with the clinical difficulties in differentiating from other hypersomnias (such as narcolepsy, delayed sleep phase syndrome, psychiatric disorder, fatigue from infections or MS, and narcotic drug abuse) makes an objective diagnostic aid highly desirable.

In search for an objective diagnostic aid there has been promising indications of a common HLA-subtype (2), but a larger material could not verify the finding (3). Decreased levels of orexin in the cerebrospinal fluid have been observed in a case during hypersomnia (4). Another case showed normal intraepisodal orexin (5).

Radiological examinations have not been able to show any specific pathology regarding brain structural changes. On the other hand, cerebral perfusion examined by single photon emission computed tomography (SPECT) has shown abnormal perfusion patterns in several case reports and case series (6–8). Discussions have been focused on SPECT as a possible tool of explaining the pathophysiology of KLS. Hypotheses regarding KLS pathophysiology include disturbances in the hypothalamic–pituitary axis, the diencephalon, and thalamo-cortical circuits (9–12).

Intraepisodal SPECT has showed a predominance of hypoperfusion in the thalami and basal ganglia (13). The finding has been consistent and a recent publication stated that intraepisodal SPECT could be used as a diagnostic aid in KLS (13, 14). In clinical praxis, however, SPECT-examinations may be difficult to perform, by several reasons. Firstly, the patient has to be admitted to a department with access to nuclear medicine facilities during an ongoing episode. Secondly, there might be problems with patient cooperation during ongoing hypersomnia. Thus, it would be of great interest to find consistent and specific abnormal perfusion patterns interepisodically.

Our group published early reports on abnormal perfusion patterns 2 weeks after an episode and also 7 years after remission. The patient showed bilateral, although predominately left-sided, fronto-temporal, and right parietal hypoperfusion. And additive finding in this patient was a persistent short-term memory dysfunction, the latter indicating persisting damage, and maybe a non-benign course of the disease (7). In a case series of four patients, two patients showed temporal and fronto-temporal hypoperfusion in between episodes or in remission (13). Other groups have shown diverse findings: in one case of posthypersomnic manic and psychotic symptoms, SPECT showed decreased perfusion in the basal ganglia, hypothalamus, and right fronto-temporal regions (15). Another case showed mesial temporal hypoperfusion both during and between hypersomnia periods (16).

In our Scandinavian group, we have consequently been performing SPECT-examinations in asymptomatic periods or after remission in order to investigate the frequency of temporal/frontal hypoperfusion and evaluate the diagnostic potential of this finding. Thus, the aim of this study is to elucidate whether measurements of brain perfusion is a practicable aid in diagnosing KLS between hypersomnia episodes and if specific findings are associated with severity of the disorder, i.e., we aimed to investigate if SPECT also could be a prognostic aid.

## Materials and Methods

We consecutively included 25 patients (11 men and 14 women) diagnosed with KLS, according to the American Academy of Sleep Medicine (2005) who had undergone SPECT as a part if the clinical investigation protocol. Patients were grouped into “non-severe KLS” and “severe KLS” based on their relation to median values (according to Arnulf et al.) of disease duration (*m* = 8 years), frequency of periods (*m* = every 3.5 months), and duration of periods (*m* = 10 days) (1). If any of these parameters exceeded the median value the patient was classified as “severe KLS.” All patients were examined in periods between hypersomnia (*n* = 12) or after remission (*n* = 9). Remission was defined as having no hypersomnia periods during the 1.5 years preceding the SPECT examination.

Single photon emission computed tomography investigations were performed using a General Electric XRT single head detector gamma camera 650 MBq 99m-Tc-HMPAO (CERETEC^®^) was administered intravenously and each patient was scanned for 32 min (64 projections in a 128 × 128 matrix acquisition). Tomographic slices were reconstructed in the transaxial, coronal, and sagittal planes using a filtered back-projection method, a Hanning prefilter with 0.8 cycles/cm cut-off frequency, and a Ramp filter without attenuation correction. The images were interpreted by an experienced specialist in nuclear medicine and compared to the standard interpretation of the method (17).

A control group was not ethically possible, due to the radioactive isotope used in the investigation.

Informed consent was obtained from all participants.

## Results

The present study shows that 48% of the investigated patients with KLS have abnormal perfusion between hypersomnia periods, as detected by SPECT. As shown in Table 1, 7 out of 16 patients with an active disease showed hypoperfusion in temporal and/or frontal regions. Patients in remission had temporal and/or frontal hypoperfusion in five out of nine cases. In total, 12 out of 25 patients showed hypoperfusion in temporal and/or frontal regions of the brain. All pathological findings in these patients were seen bilaterally or on the right side.

**Table 1:** **SPECT findings**.

	Temporal hypoperfusion	Fronto-temporal hypoperfusion	Frontal hypoperfusion	Normal perfusion	Bilateral hypoperfusion	Left hypoperfusion	Right hypoperfusion	Men	Women
Active non-severe	1		1	3	2			2	3
Active severe	4	1		6	1	2	2	4	7
Remission non-severe	2	2	1	2	4		1	3	4
Remission severe				2				2	

In patients categorized as severe, 5 out of 13 patients showed temporal and/or frontal hypoperfusion. In the non-severe patients, 7 out of 12 showed hypoperfusion in the temporal and/or frontal regions. No further regions of abnormal perfusion could be detected in any patient.

**Figure 1:**
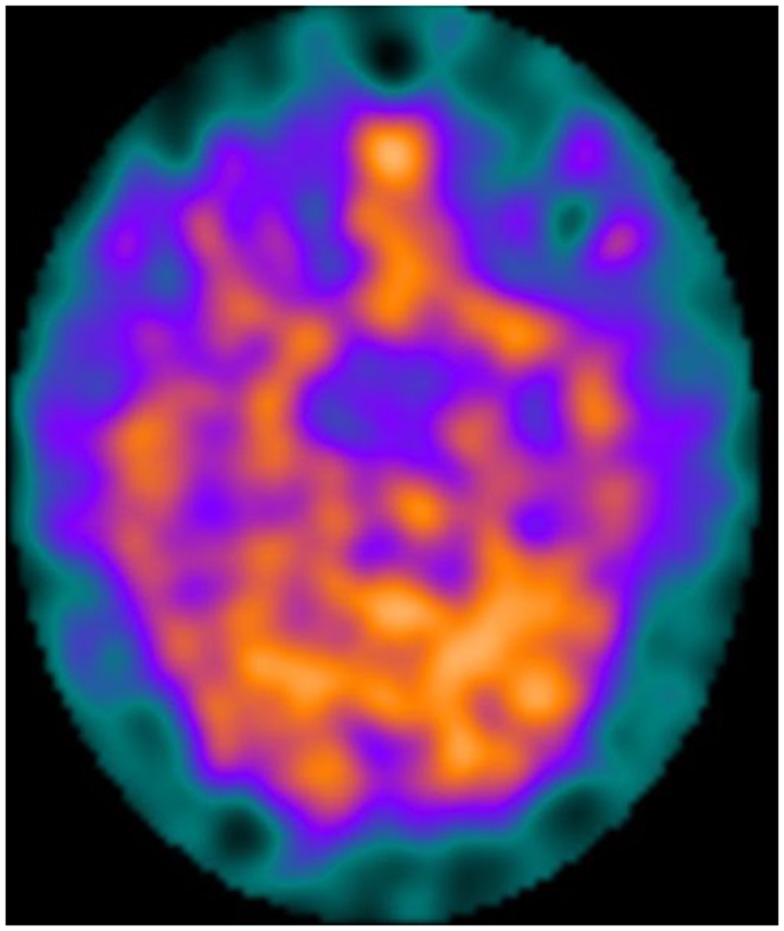
**A female patient with active Kleine–Levin syndrome with a normal SPECT perfusion pattern**.

**Figure 2:**
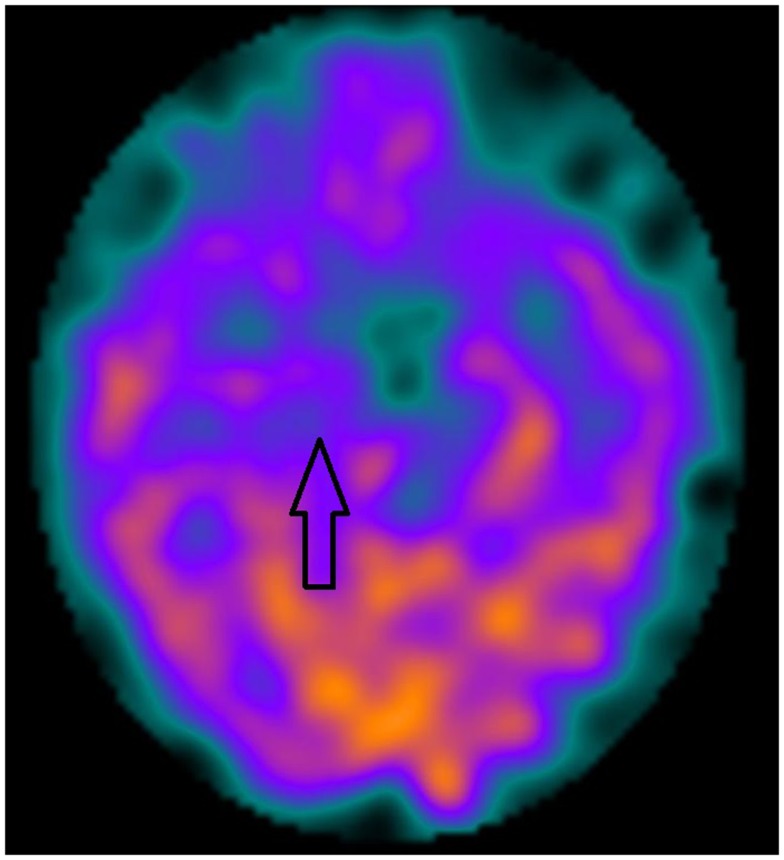
**A male patient with active Kleine–Levin Syndrome with medial temporal unilateral hypoperfusion (arrow)**.

Figures 1 and 2 show two KLS-patients with active disease, who have normal perfusion (Figure 1) and temporal hypoperfusion (Figure 2) on SPECT-examinations.

## Discussion

This study focuses on frontal/temporal hypoperfusion between episodes or after remission, in relation to results from other groups focusing on thalamic/diencephalic hypoperfusion during episodes. With 48% of patients showing non-episodal (interepisodal or post-remission) hypoperfusion in temporal or fronto-temporal regions it is clearly of interest to investigate this finding in more depth and possibly relate to different characteristics of the individual patients (3, 12, 15, 18). A sensitivity of 48%, however, is not considered strong enough to use interepisodal SPECT as an isolated tool in the diagnostic process. A combination of SPECT and specific symptomatology could possibly lead to a combined higher sensitivity than with SPECT alone. The sensitivity is to be compared to intraepisodal findings where hypoperfusion in the thalami and the basal ganglia is a consistent finding (13). For an experienced sleep neurologist, however, SPECT might be a helpful complement to the clinical diagnostic process, for instance if symptoms are suspected to be functional whereas a pathological perfusion pattern may support an organic explanation.

Another finding is that in patients that have undergone remission, 56% showed persisting hypoperfusion, supporting a chronic damage, not yet correlated to clinical follow up. As temporal and fronto-temporal regions are involved in working memory performance, the findings are consistent with our earlier findings of persisting cognitive deficits in the working memory domain (8, 13, 15, 19). The latter could be indicative of a course of the disorder that is not benign.

In this study, no obvious conclusions can be drawn regarding the prognostic value of SPECT, although more patients in remission have areas of hypoperfusion than the group in total. Additional studies of the cognitive function of these patients will be analyzed in the future to further elucidate the correlation to prognosis. A recent review of the literature found that only small series of brain perfusion imaging has been published, but in these studies, there are indications of persistent temporal and/or frontal hypoperfusion even after remission (3, 13, 15). Being the largest published material on interepisodal SPECT, the present study supports previously published results of fronto-temporal hypoperfusion persisting beyond the active phases of the disease. Patients in remission with abnormal perfusion showed predominantly right-sided or bilateral hypoperfusion.

The present study has some obvious limitations. The heterogeneous group of patients, regarding active/remission, symptomatology, and disease duration, might clearly be confounding as perfusion patterns theoretically could be changing over time and be associated to specific symptom patterns. SPECT findings were not analyzed in relation to specific clinical symptoms or findings in neuropsychological tests, something that might be of interest in future investigations. The absence of a matched control group, obviously, is a major limitation, though a limitation shared with other studies in the field due to the radioactive isotope used in SPECT (14).

## Conclusion

This study confirms results in previously published case reports and we suggest that SPECT could be an additive diagnostic tool in KLS. Regarding the low sensitivity of 48%, interepisodal SPECT must be used in a setting of vast clinical experience of the diagnostic criteria and clinical variety of the disorder. Sensitivity of SPECT seems lower interepisodically than intraepisodically.

## Conflict of Interest Statement

The authors declare that the research was conducted in the absence of any commercial or financial relationships that could be construed as a potential conflict of interest.
